# The indispensability of methyltransferase-like 3 in the immune system: from maintaining homeostasis to driving function

**DOI:** 10.3389/fimmu.2024.1456891

**Published:** 2024-10-02

**Authors:** Mingfu Zhang, Zhixian Gou, Yi Qu, Xiaojuan Su

**Affiliations:** ^1^ Department of Pediatrics/Key Laboratory of Birth Defects and Related Diseases of Women and Children (Ministry of Education), West China Second University Hospital, Sichuan University, Chengdu, China; ^2^ Department of Pediatrics, School of Clinical Medicine & the First Affiliated Hospital of Chengdu Medical College, Chengdu, China; ^3^ National Health Commission Key Laboratory of Chronobiology, Sichuan University, Chengdu, China

**Keywords:** methyltransferase-like 3, hematopoietic stem/progenitor cells, innate immune cells, adaptive immune cells, immune homeostasis

## Abstract

Methyltransferase-like 3(METTL3), recognized as the primary N^6^-methyladenosine methyltransferase, influences cellular functions such as proliferation, migration, invasion, differentiation, and fate determination by regulating gene expression post-transcriptionally. Recent studies have highlighted the indispensability of METTL3 in various immune cells such as hematopoietic stem/progenitor cells, innate immune cells (monocytes, macrophages, dendritic cells), and adaptive immune cells (thymic epithelial cell, T cells, natural killer cells). However, a comprehensive summary and analysis of these findings to elucidate the relationship between METTL3 and the immune system is yet to be undertaken. Therefore, in this review, we systematically collate reports detailing the mechanism underlying the role of METTL3 in regulating various immune processes and examine the modification of METTL3 and its potential implications. This review suggests that METTL3 plays an essential role in the immune system, ranging from maintaining homeostasis to regulating functions. Collectively, this review provides a comprehensive analysis of the relationship between METTL3 and the immune system, serving convenient researchers to understand the frontiers of immunological research and facilitate future clinical applications.

## 1 Introduction

Functioning as the primary safeguard against infectious agents in the human body, the immune system encompasses various cells, molecules, tissues, and organs (1, 2). Immune cells originate as precursors in the bone marrow and undergo transformative stages at various sites throughout the body to reach maturity (2–4). Distributed uniquely throughout the body, each type of cell and molecule serves specific functions in response to infections, intercellular communication, and problem-solving, employing various mechanisms, including inhibiting tumor growth, initiating tissue repair processes, and regulating the immune system to promote overall health (5–7). Therefore, a comprehensive understanding of the intricate mechanisms underlying the functional network of the immune system is valuable for researchers to address immunological problems, spanning from infections to cancers. Currently, epigenetics represents the cutting-edge approach for mechanism study of gene expression (8, 9).

RNA N^6^-methyladenosine (m^6^A) modification is a distinctive mechanism of epigenetic regulation, which has been extensively reported to play pivotal roles in modulating cellular biological functions, including proliferation, differentiation, and fate determination, alongside other biological activities (10–15). m^6^A modification intricately regulates the splicing, translocation, stability, and translation of RNA through dynamic and reversible interactions with m^6^A-specific regulatory proteins, such as methyltransferases, demethylases, and binding proteins (16–23). The interplay between these writers, erasers, and readers of m^6^A marks ensures a finely-tuned regulation of RNA metabolism. Among these m^6^A-specific regulatory proteins, methyltransferase-like 3 (METTL3) is the most indispensable and contributing component, which is not only the first identified but also possesses catalytic activity in RNA m^6^A modification (24). A growing number of studies have demonstrated the involvement of METTL3 in the immune system, highlighting its crucial role in regulating various physiological events (25, 26). Although the roles and mechanisms of METTL3 in the immune system have been extensively investigated, reviews that systematically interpret their relationships are lacking.

Therefore, this review focuses on summarizing findings from studies that report the roles and mechanisms of METTL3 in immune cells, thereby highlighting the prospective implications of METTL3 in the immune system alongside future research directions. Furthermore, this review provides an updated compilation of the role of METTL3 in the immune system, serving to facilitate future clinical applications and provide researchers with insights into the frontiers of immunological research.

## 2 Detailed overview of METTL3

METTL3, also known as methyltransferase-like 3, is a key component of the m^6^A methyltransferase complex (17). This enzyme plays a pivotal role in the post-transcriptional modification of RNA, influencing various physiological processes and pathophysiological conditions (27, 28). Structurally, METTL3 forms a heterodimer with METTL14, another methyltransferase-like protein, to execute its catalytic functions. The METTL3-METTL14 complex is further stabilized by Wilms’ tumor 1-associating protein (WTAP), which enhances its methyltransferase activity (17). The structural uniqueness of METTL3 lies in its catalytic domain, which is responsible for transferring the methyl group from S-adenosylmethionine to the adenosine base in RNA (17). This catalytic domain is highly conserved and distinct in METTL3, giving it an edge over other m^6^A-specific regulatory proteins. Moreover, METTL3’s role extends beyond its enzymatic activity. It has been implicated in various cellular processes, including mRNA stability, splicing, translation, and nuclear export (29). For instance, METTL3-mediated m^6^A modification is crucial for the proper splicing of pre-mRNA, as it recruits splicing factors to the methylated sites (29). Additionally, METTL3 influences mRNA stability by interacting with m^6^A readers such as YTHDF proteins, which recognize and bind to m^6^A-modified transcripts, leading to either their stabilization or degradation (29). In conclusion, METTL3 is a central player in the m^6^A RNA modification landscape, with unique structural and functional attributes that distinguish it from other m^6^A regulatory proteins. Its structural configuration, interaction with METTL14, and broad impact on RNA metabolism underscore its significance in immune cell regulation.

## 3 Relationship between METTL3 and the immune system

The immune system, which combats microbes, is primarily divided into two distinct reactions: innate and adaptive immunity (30). Innate immunity is a natural defense mechanism that confers protection from birth onwards (30). For example, the skin acts as an effective barrier for the body, protecting against bacteria, viruses, and other disease-causing pathogens (31). Conversely, adaptive immunity, which is acquired after birth, is honed over a lifetime through interactions with infectious agents or vaccinations (31). Both types of immune responses require specific cells, which can be derived from stem cells, to execute their functions.

### 3.1 Roles and mechanisms of METTL3 in hematopoietic stem cells

Hematopoietic stem/progenitor cells (HSPCs), the predominant progenitor cells located in the bone marrow, can generate all types of blood cells, including those of myeloid and lymphoid lineages, while also possessing self-renewal capacity (32).

Recent studies have highlighted the crucial role of METTL3-mediated RNA m^6^A modification in regulating HSPC function. For instance, during zebrafish embryogenesis, deletion of METTL3 in embryos by using CRISPR–Cas9 system reduces m^6^A levels by promoting the decay of the arterial endothelial genes *notch1a* and *rhoca* in a YTH N^6^-methyladenosine RNA binding protein 2 (YTHDF2)-dependent manner, which subsequently activates the Notch signaling to block HSPC production (33). Notably, METTL3-mediated m^6^A modification functions in the process of endothelial-to-hematopoietic transition to specify the earliest HSPCs. Additionally, by using the hematopoietic-specific *Vav*-Cre^+^-*Mettl3^fl/fl^* mice model, Gao et al. (34) demonstrated that specific deletion of *METTL3* in the liver of murine fetuses results in hematopoietic deficiency and increases perinatal mortality. METTL3 activates the Mavs or RNase L signaling pathways, precipitating an aberrant innate immune response. However, under pathological conditions of acute myeloid leukemia (AML), a converse function is reported, wherein the levels of METTL3 are elevated compared to those in healthy HSPCs (35). Intriguingly, shRNA-mediated silencing of METTL3 in HSPCs of human AML promotes HSPC differentiation by increasing p-AKT levels and facilitates apoptosis by promoting the mRNA translation of *c-MYC*, *BCL2*, and *PTEN* while inhibiting proliferation. Therefore, the detrimental role of METTL3 in the HSPCs of patients with AML indicates its potential as a therapeutic target.

In summary, the available studies highlight the pivotal role of METTL3 in regulating HSPC function. Although relatively elevated expression levels facilitate physiological differentiation, they may also contribute to pathological events.

### 3.2 Roles and mechanisms of METTL3 in innate immune cells

Common myeloid progenitor stem cells, which reside in the bone marrow, serve as precursors to generate various innate immune cells, including but not limited to neutrophils, eosinophils, basophils, mast cells, monocytes, dendritic cells (DCs), and macrophages (36). These primary responders promptly act against infections, such as inflammation (30). Therefore, maintaining their normal function is essential for an effective innate immune response. Given that METTL3 plays a crucial role in regulating the biological functions of various cells, numerous studies have reported its significance in these innate immune cells (37, 38). Innate lymphoid cells (ILCs) can quickly switch from a quiescent state to an active state and rapidly produce effectors that provide critical early immune protection (39). In *R26* ^Cre^ *Mettl3* ^fl/fl^ mice model, Zhang et al. (40) showed that deletion of METTL3 significantly diminishes ILC2 proliferation, migration, and effector cytokine production and results in impaired anti-helminth immunity, whereas exerts minimal influence on ILC homeostasis or the cytokine-driven responses of ILC1 or ILC3 subtypes. Importantly, the gene encoding the transcription factor *Gata3* is highly m^6^A methylated in ILC2. Moreover, demethylation of m^6^A weakens *Gata3* mRNA stability and impairs Gata3 upregulation and ILC2 activation. Notably, METTL3-mediated m^6^A modification is essential for ILC state transition and immune response.

#### 3.2.1 METTL3 triggers monocyte inflammation

**Figure 1:**
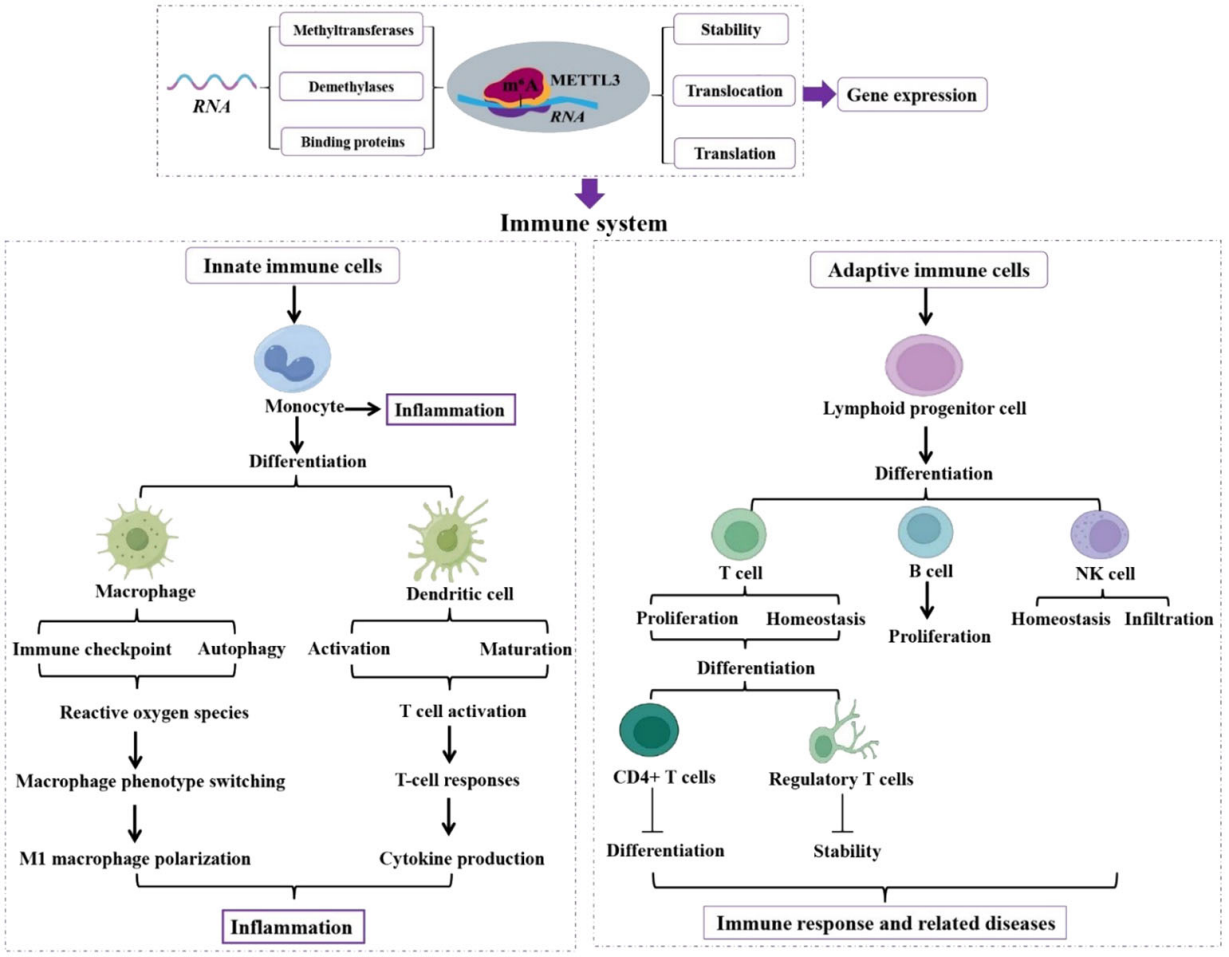
Role of METTL3 in innate and adaptive immune cells. Functions of METTL3 in innate and adaptive immune cells are analyzed, which is essential in regulating immune system homeostasis and function. Methyltransferase-like 3 (METTL3). N^6^-methyladenosine (m^6^A). Natural killer (NK).

**Figure 2:**
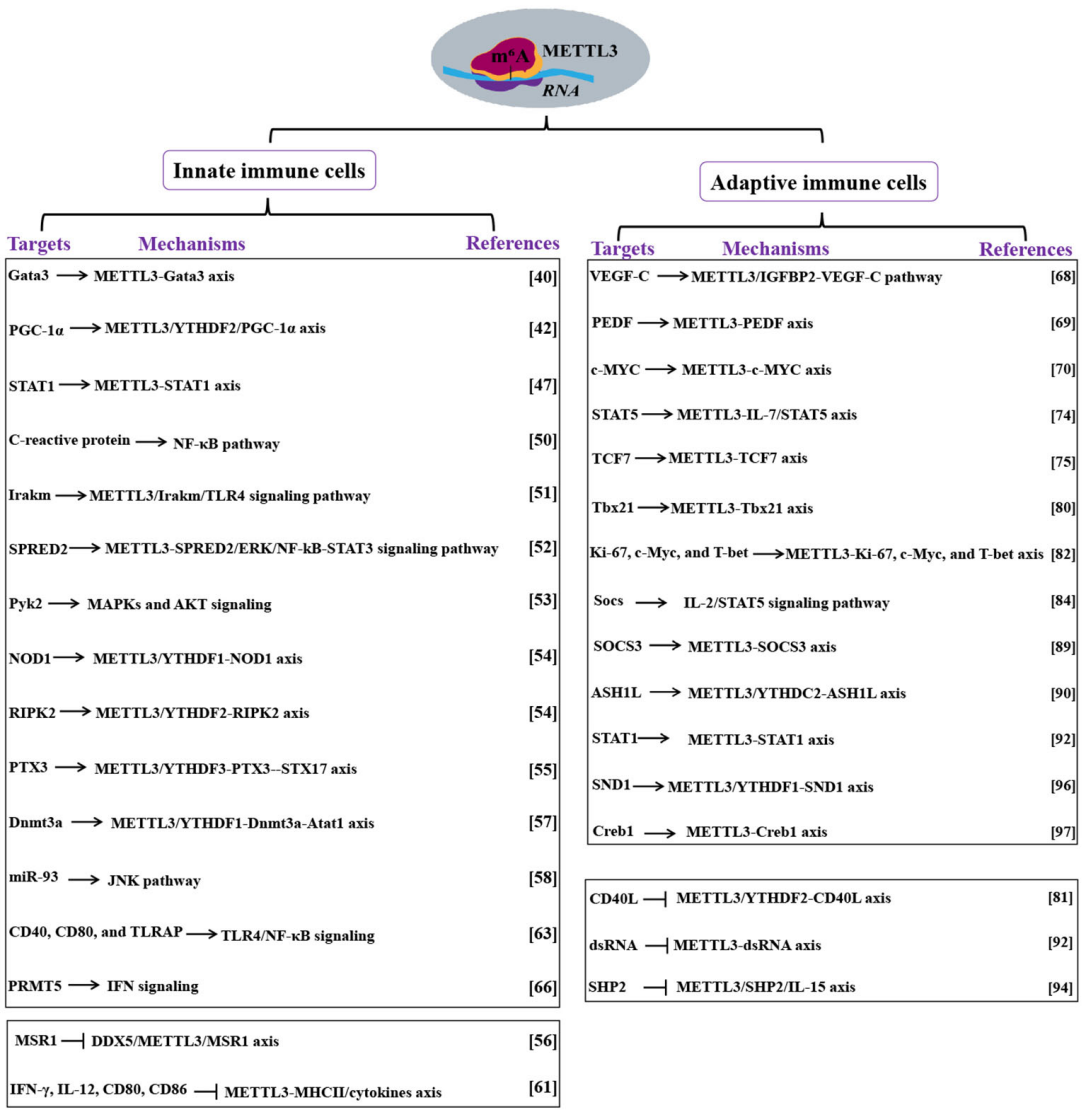
Mechanism of METTL3 actions in immune cells. Targets and mechanisms of METTL3 in innate and adaptive immune cells are analyzed. Methyltransferase-like 3 (METTL3). N^6^-methyladenosine (m^6^A). Proliferator-activated receptor gamma coactivator 1-alpha (PGC-1α). YTH N6-methyladenosine RNA binding protein (YTHDF). Signal transducer and activator of transcription (STAT). Toll-like receptor 4 (TLR4). Sprouty related EVH1 domain containing 2(SPRED2). DNA methyltransferase 3A (Dnmt3a). Alpha-tubulin acetyltransferase 1 (Atat1). MicroRNA-93 (miR-93). TLR4 signaling adaptor (TIRAP). Protein arginine methyltransferase 5 (PRMT5). Interferon (IFN). DEAD box helicase 5(DDX5). Macrophage scavenger receptor 1(MSR1). Interferon-gamma (IFN-γ). Interleukin (IL). Major histocompatibility complex (MHC). Vascular endothelial growth factor (VEGF). Signal transducer and activator of transcription (STAT). Interleukin (IL). Absent, small, or homeotic-like 1 (ASH1L). YTH domain-containing 2 (YTHDC2). Staphylococcal nuclease and Tudor domain-containing protein 1 (SND1). Double-stranded RNA (dsRNA). CD40 ligand (CD40L).

Monocytes, which reside primarily in the bloodstream and tissues, can manipulate and respond to stimuli (41). Zhang et al. (42) reported a mechanism of METTL3-mediated RNA m^6^A modification that triggers monocyte inflammation in response to oxidized low-density lipoprotein (ox-LDL). This intricate process involves the coordinated actions of METTL3 and YTHDF2, which collaborate to promote peroxisome proliferator-activated receptor gamma coactivator 1-alpha (*PGC-1α*) mRNA degradation, leading to decreased PGC-1α expression and culminating in an escalated inflammatory response. In addition to downregulating PGC-1α expression, this coordination also suppresses the expression levels of NDUFC2, as well as ATP production and oxygen consumption rate. Consequently, this cascade induces the accumulation of reactive oxygen species (ROS) in both the cellular and mitochondrial compartments, thereby enhancing pro-inflammatory cytokines in inflammatory monocytes. Therefore, the METTL3/YTHDF2/PGC-1α axis regulates monocyte-mediated inflammatory responses (**Figures 1**, **2**).

#### 3.2.2 METTL3 functions in macrophage phenotype switch

Macrophages are crucial regulators of the innate immune and are also the major producers of inflammatory cytokines once activated by pathogen-associated molecular patterns (43). Monocytes can differentiate into distinct macrophage phenotypes with remarkable phagocytic and bacterial degradation capabilities (44). Upon activation by an external stimulus, monocytes collaborate with macrophages to induce an immune response by signaling other immune cells (45).

In addition to regulating the inflammatory response of monocytes, METTL3 is also crucial for maintaining macrophage function (46). Liu et al. (47) found an upregulation of METTL3 during mouse M1 macrophage polarization. Moreover, METTL3 overexpression not only facilitates M1 macrophage polarization but also attenuates M2 polarization. Conversely, METTL3 downregulation leads to contrasting effects. Mechanically, METTL3 functions by methylating the signal transducer and activator of transcription 1 (*STAT1*) mRNA at its coding sequence and 3’-untranslated regions to enhance *STAT1* mRNA stability and subsequently upregulates STAT1 expression, which controls M1 macrophage polarization. Therefore, METTL3 promotes macrophage polarization toward the M1 pro-inflammatory phenotype by targeting *STAT1* mRNA, rendering it a promising anti-inflammatory target in inflammation-induced diseases. For example, rheumatoid arthritis (RA) is strongly associated with monocyte-macrophage inflammation (48, 49). Wang et al. (50) demonstrated that METTL3 expression is significantly upregulated in patients with RA and is positively correlated with C-reactive protein and erythrocyte sedimentation rate, which are markers of RA. METTL3 downregulation alleviates lipopolysaccharide (LPS)-induced inflammatory response in M1 macrophages. Moreover, the impact of METTL3 on LPS-induced macrophage inflammation is closely associated with the NF-κB signaling pathway. Notably, METTL3 promotes M1 pro-inflammatory phenotype activation in macrophages via the NF-κB pathway, highlighting the pivotal role of METTL3 in driving macrophage phenotype switch in RA and its potential utility as a biomarker for this condition.

Similarly, Tong et al. (51) showed that *Mettl3* knockdown in macrophages decreases tumor necrosis factor-α (TNF-α) expression upon LPS stimulation *in vitro*. Furthermore, mice with a specific knockdown of *Mettl3* in macrophages (*Mettl3* ^fl/fl^; *Lyzm*-^Cre^ mice model) are more susceptible to bacterial infection and exhibit faster tumor growth. METTL3 deregulation increases the stability and expression of *Irakm*, a negative regulator of toll-like receptor 4 (TLR4) signaling, which subsequently inhibits macrophage-mediated innate immune response to inflammation by deactivating TLR4 signaling. These findings suggest that the METTL3/Irakm/TLR4 signaling pathway represents a novel mechanism for regulating macrophage-mediated innate immune responses, highlighting its clinical applications in immunotherapy. Yin et al. (52) demonstrated that the specific knockdown of *Mettl3* in the myeloid cells of mice (crossed *Mettl3* ^fl/fl^ mice with *Lyz*-^cre^ mice to delete *Mettl3*) leads to tumor progression by promoting the accumulation of M1/M2-like tumor-phenotype macrophage and facilitating the infiltration of regulatory T cell (Treg) into tumors. More importantly, the therapeutic efficacy of programmed cell death 1 is compromised by the *Mettl3* downregulation, which obstructs the immune checkpoint. This downregulation decreases sprouty-related EVH1 domain containing 2 (*SPRED2*) translation, mediated by YTHDF1, thereby activating the ERK pathway and enhancing NF-kB and STAT3 expression. Therefore, METTL3 collaborates with YTHDF1 to promote tumor progression and attenuate the efficacy of tumor therapy by targeting the SPRED2/ERK/NF-kB-STAT3 signaling pathway, highlighting the clinical possibilities of using METTL3 as a target in tumor immunotherapy.

However, in contrast, Cai et al. (53) found that METTL3 attenuates LPS-induced proinflammatory pathways and the ROS generation process. Mechanically, METTL3-YTHDF2 enhances the *Pyk2* mRNA stability, which consequently activates the MAPKs and AKT signaling to promote the generation of proinflammatory cytokines and ROS. The total levels of m^6^A and METTL3 are decreased in LPS-stimulated macrophages. *Mettl3* knockdown significantly upregulates proinflammatory cytokines (TNF-α, IL-6, and NO), which enhances the mRNA stability and expression of *NOD1* and *RIPK2* by interacting with YTHDF1 and YTHDF2, respectively (54). All findings suggest that METTL3 promotes the LPS-induced inflammatory response in macrophages through mediating m^6^A modification on *NOD1* and *RIPK2*. Furthermore, METTL3 has a low expression level in monocyte-derived macrophages from patients with childhood allergic asthma (55). Conditional knockout of *METTL3* in myeloid cells (*Mettl3* ^fl/fl^ *Lyz2* ^Cre+^) enhances Th2 cell response and aggravates allergic airway inflammation by facilitating M2 macrophage activation (55). A low level of METTL3 suppresses *PTX3* mRNA degradation and induces PTX3 expression in a YTHDF3-dependent manner. Furthermore, the METTL3/YTHDF3-PTX3 axis contributes to autophagy maturation in macrophages by modulating STX17 expression and thereby promoting M2 macrophage activation. Notably, METTL3 promotes M2 macrophage activation via regulating the PTX3-STX17 axis, thereby identifying METTL3 as a potential target for controlling allergic asthma.

Macrophages also possess crucial non-immunological functions, such as clearing cellular debris and recycling of deceased cells (such as red blood cells) (45). These “housekeeping” activities occur independently of immune response activation. Zhao et al. (56) demonstrated that ox-LDL-induced DEAD-box helicase 5 (DDX5) upregulation facilitates lipid uptake in macrophages, which is not dependent on either the MAPK or NF-κB pathway. Mechanistically, DDX5 reduces METTL3 modification on macrophage scavenger receptor 1(*MSR1*) mRNA to maintain *MSR1* stability. Overall, these findings suggest that ox-LDL-induced lipid uptake in macrophages by targeting the DDX5/METTL3/MSR1 axis. Besides, Yin et al. (57) found that *Mettl3* deletion in monocyte-derived macrophages (*Mettl3* ^fl/fl^ *Lyz2* ^Cre/-^) reduces the m^6^A modification on DNA methyltransferase 3A (*Dnmt3a*) mRNA and impairs YTHDF1-mediated *Dnmt3a* translation, which in turn interacts with alpha-tubulin acetyltransferase 1 (*Atat1*) promoter to maintain its expression, improving cognitive function in an amyloid beta (Aβ)-induced Alzheimer’s disease (AD) mouse model. Therefore, METTL3 promotes the migration of monocyte-derived macrophages and Aβ clearance by regulating the Dnmt3a-Atat1 axis, ultimately alleviating AD, highlighting that targeting METTL3 is a promising target for AD treatment in the future. Furthermore, the aberrant cross-talk between macrophages and bronchial epithelial cells is essential for the degradation of elastin which contributes to emphysema, in which METTL3 plays a critical role (58). Xia et al. (58) established that METTL3-mediated m^6^A modification promotes the production of excess mature microRNA-93 (miR-93) in bronchial epithelial cells, which are then transferred from bronchial epithelial cells into macrophages. In macrophages, miR-93 activates the JNK pathway by targeting dual-specificity phosphatase 2, which elevates matrix metalloproteinase 9 and 12 and induces *elastin* degradation, leading to emphysema (58). Notably, METTL3 is critical for the aberrant cross-talk of epithelium-macrophages in emphysema and, thereby may be used in clinical diagnosis for emphysema (**Figures 1**, **2**).

Notably, METTL3-mediated RNA m^6^A modification plays a crucial role in regulating the intricate functions of macrophages, as well as macrophage-related diseases, suggesting its role in disease treatment.

#### 3.2.3 METTL3 contributes to DC activation and maturation

Monocytes can differentiate into DCs, which are crucial antigen-presenting cells (APCs) (59). Similar to DCs, APCs play a vital role in breaking down large molecules into “readable” fragments or antigens recognizable by adaptive B or T cells (59). However, T cell activation is contingent not only upon antigens but also upon the correct major histocompatibility complex (MHC) II expressed on the surface of APCs (60). The MHC, which acts as a checkpoint, aids immune cells in distinguishing self from non-self (60). Wu et al. (61) demonstrated that *METTL3* knockdown in DCs reduces the level of MHCII costimulatory molecules (CD80, CD86) and DC-related cytokines (IFN-γ, IL-12) and inhibits its ability to activate T-cell proliferation, consistent with the characteristics of tolerogenic DCs. Moreover, knockdown of *METTL3* in DCs by using the lentiviruses expressing METTL3-specific shRNA, results in Th1/Th2 immune tolerance following mouse heart transplantation and prolongs allograft survival (61). Notably, METTL3 plays an essential role in DC activation and optimal immune function. Another study showed that exosomes derived from *METTL3*-deficient DCs effectively prevent immune rejection in a mouse cardiac allograft model (62). Furthermore, Wang et al. (63) reported that *Mettl3* knockdown impedes the phenotypic and functional maturation of DCs. The METTL3-mediated m^6^A methylation facilitates the translation of CD40, CD80, and TLRAP (a TLR4 signaling adaptor) in DCs to promote T cell activation and reinforces cytokine production induced by TLR4/NF-κB signaling. These findings underscore the indispensability of METTL3-mediated m^6^A modification in enhancing DC activation and maturation, as well as T-cell responses, which are achieved through stimulating the translation of specific immune transcripts.

Blastic plasmacytoid DC neoplasm (BPDCN) is a rare and aggressive hematologic malignancy with poor clinical outcomes (64). BPDCN is highly associated with the low level of protein arginine methyltransferase 5 (PRMT5) (65). Rethnam et al. (66) demonstrated that PRMT5 inhibition reduces METTL3 expression, which then activates the interferon (IFN) signaling. This increase in IFN signaling attenuates the sensitivity of METTL3 silencing to PRMT5 inhibition. Notably, the cellular function of METTL3-mediated RNA m^6^A modification is also affected by PRMT5 inhibition (66). Overall, these findings indicate that METTL3 and the IFN pathway regulate the response to PRMT5 inhibition in BPDCN, implicating the involvement of METTL3 in BPDCN (**Figures 1**, **2**).

In conclusion, maintaining a relatively elevated level of METTL3 is indispensable for the normal function of innate immune cells. Furthermore, targeting METTL3 may be an effective therapeutic approach against diseases caused by immune cell dysfunction.

### 3.3 Roles and mechanisms of METTL3 in adaptive immune cells

The adaptive immune cells, including B, thymic epithelial, and natural killer (NK) cells, collectively known as lymphocytes, originated from common lymphoid progenitor stem cells (67). When adaptive immune cells in the lymph nodes recognize microbial fragments from a distant region, they initiate an active immune response by activating, replicating, and exiting the lymph nodes to circulate and combat the pathogen (67). METTL3-mediated RNA m^6^A modification has been reported to be involved in regulating the function of adaptive immune cells, thus modulating the adaptive immune response.

#### 3.3.1 METTL3 switches the phenotype of thymic epithelial cells

Zhou et al. (68) demonstrated that adipose-derived mesenchymal stem cells expedite lymphatic endothelial cell proliferation, migration, and lymphangiogenesis via the METTL3/IGFBP2/VEGF-C pathway. Another study reported that METTL3 overexpression promotes the proliferation of diffuse large B-cell lymphoma cell lines by regulating the m^6^A levels of *PEDF* (69). In this study, the expression level of METTL3 is elevated in thymic epithelial tumors (TET), contributing to the development of the TET phenotype by stimulating cell proliferation. In addition, by relocating lncRNA MALAT1 in TET cells, METTL3 enhances the translation rate of *c-MYC* (70). These studies demonstrate that the elevated levels of METTL3 contribute to the impairment of progenitor cells in the adaptive immune response, leading to the manifestation of the corresponding diseases (**Figures 1**, **2**).

#### 3.3.2 METTL3 maintains the normal functions of T-cell

T cells, which are usually classified into CD4+ or CD8+ cells, perform various functions, such as eliminating infected cells and activating or recruiting other immune cells (71). T-cell activation is a highly regulated process, which is modulated by various immune regulatory proteins including cytokines, surface receptors, and co-stimulatory proteins (72, 73). Li et al. (74) demonstrated that silencing *Mettl3* in mouse T cells (*Mettl3* ^fl/fl^; *CD4* ^Cre^) disrupts cell homeostasis and differentiation. METTL3 regulates T cell homeostasis and differentiation by modulating the IL-7-mediated STAT5 axis. Specifically, METTL3 downregulation in undifferentiated T cells increases the expression of Socs1, Socs3, and Cish, inhibits IL-7-induced STAT5 activation, and impairs T cell proliferation and differentiation. Additionally, Yao et al. (75) discovered that the specific knockdown of *Mettl3* in CD4+ T cells impede the normal differentiation of T follicular helper (TFH) cells in mice by inhibiting the expression of TCF7, a crucial TFH signature gene, suggesting the indispensable role of METTL3 in regulating the differentiation of TFH cells.

As a key component of the adaptive immune system, CD8+ T cells protect the body against various intracellular pathogens and clear autologous malignant cells (76). Once activated, naive CD8+ T cells (Tn) proliferate rapidly and differentiate into cytotoxic effector cells (Te) with the capacity to produce proinflammatory cytokines such as IFN-γ and TNF-a and expressing cytolytic molecules such as perforin and granule enzymes (77). Effector CD8+ T cells (Tm) are ascertained as a heterogeneous population that expresses high levels of interleukin 7 receptor (78). Notably, CD8+ T cells at different states (Tn, Te, and Tm) show stage-specific molecular, phenotypic, and functional characteristics (79). Guo et al. (80) demonstrated that METTL3 deletion specifically in CD8+ T cells weaken its effector cell expansion and terminal differentiation by stabilizing the *Tbx21* transcript in an m^6^A-dependent manner, subsequently affecting memory formation and the secondary response of CD8+ T cells. Notably, METTL3 regulates the stage transition of CD8+ T cells by targeting *Tbx21*, underscoring the importance of METTL3 in controlling CD8+ T cell functions.

m^6^A has been regarded as a novel regulator for CD40 ligand (CD40L) expression in human CD4+ lymphocytes (81). METTL3 promotes *CD40L* mRNA degradation and affects the expression of CD40L via YTHDF2 recognizing the specific sequences on the *CD40L* mRNA (81). Therefore, CD40L expression in human primary CD4+ T lymphocytes is regulated via the METTL3/YTHDF2-m^6^A modification. This study elucidates a new regulatory mechanism in CD4+ T cell activation that can be used to modulate T cell responses in patients with immune-related diseases. Evidence also showed that the alloreactive CD4+ T cells play a central role in allograft rejection. Li et al. (82) found that graft-infiltrating CD4+ T cells show high levels of m^6^A. Importantly, METTL3 inhibition reduces m^6^A levels, inhibits T-cell proliferation, and suppresses effector differentiation of polyclonal CD4+ T cells. Inhibition of METTL3 in alloreactive CD4+ T cells suppress T-cell proliferation and T helper type 1 cell differentiation, arrests the cell cycle in the G0 phase and elevates cell apoptosis. Moreover, these impaired T-cell responses are accompanied by reduced expression levels of Ki67, c-Myc, and T-bet. It noted that a low level of METTL3 impairs the T-cell effector program and suppresses alloreactive CD4+ T-cell effector function and differentiation by reducing the expression of Ki-67, c-Myc, and T-bet. Therefore, targeting METTL3 in CD4+ T cells represent an attractive therapeutic approach to prevent allograft rejection. Furthermore, Treg cells are a subset of CD4+ T cells that suppress the activity of other T cells, thereby preventing deleterious immune activation and maintaining tolerance to self-antigens (83). Tong et al. (84) demonstrated that knockout of *Mettl3* in Treg cells of mice (*Mettl3* ^f/f^; *Foxp3^Cre^*) results in several adaptive immune responses such as autoimmune disease. These findings suggest that the inhibitory function of Treg cells is systematically compromised in the absence of m^6^A RNA modification. The loss of METTL3/m^6^A in Treg cells leads to the upregulation of *Socs* mRNA levels, which subsequently deactivate the IL-2/STAT5 signaling pathway, a crucial factor for maintaining Treg cell function and stability. These studies suggest that the elevated level of METTL3 is necessary for preserving the normal function of T-cells, which is essential for adaptive immune responses.

T helper 17 (Th17) cells play a pivotal role in host defense and autoimmunity, which commonly consist of two distinct subsets: non-pathogenic and pathogenic Th17 cells (85, 86). Nonpathogenic Th17 cells are generated in the presence of TGF-β, IL-6, IL-17, and IL-10 (87). In contrast, IL-23 alone or together with IL-6 induces highly pathogenic Th17 cells that express signature genes including IL-17A, IL-17F, IL-23R, IL-1R, and granulocyte-macrophage colony-stimulating factor (88). METTL3 silencing reduces IL-17A and CCR5 expression by enhancing *SOCS3* mRNA stability in pathogenic Th17 cells, disrupts Th17 cell differentiation and infiltration, and ultimately attenuates experimental autoimmune encephalomyelitis development (89). Therefore, the METTL3-SOCS3 axis regulates pathogenic Th17 cell functions, as well as implies METTL3 as a potential therapeutic target for pathogenic Th17 cell-mediated autoimmune disease. In contrast, Zhao et al. (90) found that METTL3 upregulation ameliorates the development of experimental autoimmune uveitis and suppresses pathogenic Th17 cell responses *in vivo* and *in vitro*. Mechanistically, METTL3/YTH domain-containing 2 (YTHDC2) promotes absent, small, or homeotic-like 1 (*ASH1L*) mRNA stability and upregulates ASH1L expression, which subsequently reduces the expression of IL-17 and IL-23R, ultimately impairs pathogenic Th17 responses. Together, these data suggest that METTL3 controls pathogenic Th17 responses, and targeting METTL3 may contribute to human autoimmune disease therapy.

Γδ T cells make key contributions to tissue physiology and immune surveillance primarily through two functional subsets, γδT1 and γδT17 (91). METTL3-mediated m^6^A methylation controls the functional specification of γδT17 and γδT1 cells by preventing endogenous double-stranded RNA (*dsRNA*) formation and promoting *STAT1* mRNA degradation, which converges to prevent the over-activation of STAT1 signaling and ensuing inhibition of γδT17 (92). Moreover, deleting *Mettl3* in γδT cells (*Mettl3* ^fl/fl^-*Cd2* ^cre^) reduces IL-17 production and ameliorates γδT17-mediated psoriasis. In summary, METTL3-mediated m^6^A methylation orchestrates *STAT1* mRNA stability and dsRNA contents to equilibrate γδT1 and γδT17 cells (**Figures 1**, **2**).

#### 3.3.3 METTL3 is essential for maintaining NK cell functions

Unlike other adaptive immune cells, NK cells possess innate and adaptive immunity characteristics and are crucial for identifying and eliminating virus-infected cells or tumor cells (93).

A low level of METTL3 and effector molecules in NK cells that infiltrate tumors has been found (94). The loss of *Mettl3* (*Mettl3* ^fl/fl^-*Ncr1* ^Cre/+^) disrupts NK cell homeostasis and impedes their infiltration and function within the tumor microenvironment. This ultimately promotes tumorigenesis and reduces the survival rates in mice. Mechanistically, METTL3 suppresses SHP2 expression, thereby inhibiting the IL-15-activated AKT and MAPK signaling pathways in NK cells. These findings demonstrate that METTL3 is essential for maintaining homeostasis and the tumor immunosurveillance function of NK cells by regulating the METTL3/SHP2/IL-15 axis.

The abnormal overexpression of METTL3 contributes to the pathogenesis and chemoresistance of NK cell-related tumors. For example, nasal-type NK/T-cell lymphoma (NKTCL) is a typical class of non-Hodgkin’s lymphoma, which is quite malignant because of its high resistance to chemotherapy (95). A high level of METTL3 is found in human NKTCL cell lines compared with normal NK cells, which stabilizes staphylococcal nuclease and Tudor domain-containing protein 1 (*SND1*) mRNA to enhance SND1 expression by depending on YTHDF1 (96). Importantly, METTL3 impairs the sensitivity of NKTCL cells to *Cisplatin* by targeting SND1. Silencing of METTL3 or SND1 suppresses tumor growth and enhances *Cisplatin* sensitivity. Therefore, METTL3 contributes to NKTCL oncogenesis and *Cisplatin* resistance. Consistently, METTL3 regulates invariant NKT (iNKT) cell development and function in an m^6^A-dependent manner. You et al. (97) showed that deletion of *Mettl3* specifically in CD4+ and CD8+ T cells disturb the stability of the *Creb1* transcript, which in turn controls the protein and phosphorylation levels of Creb1, inhibiting iNKT cell proliferation, differentiation, and cytokine secretion, ultimately causing defects in B16F10 melanoma resistance. Importantly, the deletion of Creb1 in CD4+ and CD8+ T cells bring about phenotypes of iNKT cells that are similar to *Mettl3* deficiency (97). Therefore, METTL3 regulates the development of iNKT cells by targeting Creb1 (**Figures 1**, **2**).

To sum up, METTL3 is required for maintaining the normal functions of adaptive immune cells, including its indispensable role in switching the phenotype of thymic epithelial cells, as well as essential for maintaining the functions of T-cells and NK cells.

## 4 Discussion

RNA m^6^A modification is the most abundant form of post-transcriptional epigenetic modification in eukaryotes (10). It functions in various cells and plays a crucial role in their biological processes (10). METTL3, as the dominant component of m^6^A, has specific catalytic capabilities that enable it to exert the effects of m^6^A modification on RNA metabolism, encompassing processing, nucleosynthesis, translation, and even decay (98). Owing to the significant advancements in m^6^A RNA sequencing, the roles and mechanisms of m^6^A modification in various normal immune system processes have been extensively explored. Owing to the wide range of investigations on METTL3 in the immune system but the notable absence of reviews, we present a comprehensive summary and analysis of these processes, which entails the functions and mechanisms of METTL3 in the immune system.

Our analysis shows that METTL3 is indispensable for regulating the homeostasis and normal functioning of the immune system. Maintaining METTL3 at relatively elevated levels is critical for the normal functioning of innate immune cells, including monocytes, DCs, and macrophages. Moreover, a similar relationship is observed between METTL3 and the adaptive immune cells, including lymphatic endothelial, T, and NK cells, indicating that relatively elevated levels of METTL3 are beneficial for their normal functioning through various targets and signaling pathways. Therefore, based on these findings, we propose that the dysregulation of METTL3 may serve as an underlying trigger for immune system disorders. Consequently, targeting METTL3 would be a promising approach for the diagnosis, prognosis, and treatment of immune cell dysfunction-related disorders. However, in HSPCs, although relatively elevated expression levels of METTL3 contribute to physiological differentiation, a conclusive stance is challenging to establish. This is because of the limited number of original publications on METTL3 in HSPCs, which present slightly contrasting conclusions. Similar conditions are also found in monocytes and thymic epithelial cells. Therefore, the formation of firm conclusions remains elusive, underscoring the need for additional studies to bridge these gaps. Besides, studies on the relationship between METTL3 and B cells are lacking. Moreover, the dearth of studies conducted in clinical settings to confirm the relevance of METTL3 in HSPCs, monocytes, thymic epithelial cells, and B, and T cells is also apparent.

This comprehensive understanding of METTL3’s role in immune regulation opens up several intriguing questions and potential research directions. One such direction is the investigation of how METTL3 interacts with the broader epigenetic landscape. The intricacies of post-translational modifications (PTMs) in METTL3 underscore the multifaceted regulation of this enzyme. PTMs such as SUMOylation, methylation, and potentially hypusine modification play critical roles in modulating its activity, stability, and subcellular localization. These modifications can influence METTL3’s interaction with other proteins and its participation in various signaling pathways, thereby impacting mRNA methylation and the subsequent gene expression profiles (99). SUMOylation, for instance, has been shown to affect the nuclear-cytoplasmic shuttling of METTL3 (100). This reversible modification can alter METTL3’s localization and, consequently, its accessibility to specific substrates within different cellular compartments, enhancing or inhibiting its methyltransferase activity depending on the cellular context and the specific SUMOylation sites involved (100). Similarly, the addition of SUMO proteins can create new interaction surfaces or mask existing ones, thereby modulating METTL3’s binding partners and functional outcomes. Hypusine modification, which is typically associated with the eukaryotic translation initiation factor 5A, involves the addition of a spermidine-derived hypusine moiety (101). If METTL3 undergoes such a modification, it could imply a novel regulatory mechanism that links translation control with mRNA methylation processes (102). This could unveil new dimensions of cross-talk between translational machinery and epigenetic regulation, highlighting the evolutionary conservation and functional diversification of hypusine modification (102). Therefore, modulating specific PTMs of METTL3 could be a strategy to alter its activity in disease contexts. For instance, aberrant methylation patterns have been implicated in various cancers, and targeting the PTMs of METTL3 could restore normal mRNA methylation and gene expression profiles (103, 104). Obviously, inhibitors or enhancers of specific PTMs could be developed to fine-tune METTL3 activity, offering precise interventions in pathological conditions where METTL3 is dysregulated. In conclusion, the PTMs of METTL3 are crucial for its functional regulation and integration into cellular signaling networks. Understanding these modifications provides a comprehensive view of METTL3’s role in gene expression and highlights potential targets for therapeutic intervention. Future research should focus on elucidating the complete PTM landscape of METTL3, identifying the upstream regulators and downstream effectors of these modifications, and exploring their implications in health and disease.

Additionally, the existing evidence underscores that METTL3 is pivotal in modulating immune cell differentiation, activation, and function. Specifically, METTL3-mediated m^6^A methylation impacts mRNA stability and translation efficiency, thereby fine-tuning the gene expression profiles essential for immune cell functions. This regulatory mechanism is crucial in maintaining immune homeostasis and responding to pathogenic challenges. Therefore, further exploring the role of METTL3 in immune regulation presents a fascinating avenue for understanding how RNA modifications influence immunological responses. For example, while the role of METTL3 in ILC2s is significant, it is essential to acknowledge that METTL3 might also influence other ILC subsets such as ILC1 and ILC3. To gain a comprehensive understanding, it is imperative to consider how METTL3 might affect cytokine production, cell differentiation, and immune responses across the different ILC subsets. Secondly, the impact of METTL3 on ILC2s in various disease conditions warrants an in-depth discussion. For example, in the context of metabolic diseases, emerging evidence suggests that ILC2s play a role in adipose tissue homeostasis and insulin sensitivity (105). Here, METTL3 might modulate these processes by influencing ILC2 proliferation and cytokine secretion, thus impacting metabolic health. Similarly, given the significant role of METTL3 in modulating macrophage function, future research should aim to uncover the context-dependent versatility of METTL3 in various tissue-specific macrophage populations. This involves utilizing advanced single-cell RNA sequencing techniques to map METTL3’s influence on macrophage heterogeneity across different organs and disease states. Additionally, exploring the interplay between METTL3 and other epigenetic modifiers could provide deeper insights into the regulatory networks governing macrophage plasticity. Moreover, the potential therapeutic implications of targeting METTL3 in macrophage-mediated diseases should be examined. For instance, manipulating METTL3 activity might offer new avenues for treating chronic inflammatory diseases, cancer, and autoimmune disorders by modulating macrophage polarization and function. Future studies could also investigate the role of METTL3 in macrophage responses to microbial infections, shedding light on how METTL3-driven epitranscriptomic modifications influence pathogen recognition and immune response activation.

Considering the elevation of METTL3 levels in various pathological conditions, it is imperative to evaluate its potential as a diagnostic marker. The utilization of METTL3 as a biomarker could revolutionize early detection and personalized treatment strategies for a multitude of diseases, particularly cancers. However, the dualistic role of METTL3 in different cellular contexts necessitates a cautious approach. Firstly, the “double-edged sword” nature of METTL3 is evident from its involvement in both tumorigenesis and tumor suppression, depending on the tissue type and the specific oncogenic pathways active. For instance, while elevated METTL3 levels have been linked to the proliferation and metastasis of AML cells, they have also been shown to inhibit the progression of glioblastoma by modulating mRNA stability and translation in a context-dependent manner (106–108). This paradoxical behavior underscores the complexity of METTL3’s function and the need for a nuanced understanding when considering it as a diagnostic tool. To mitigate these pitfalls, a multi-faceted approach is necessary. Combining METTL3 expression data with other molecular markers and clinical parameters could enhance diagnostic accuracy and provide a more comprehensive understanding of the disease state. Advanced techniques like single-cell RNA sequencing and spatial transcriptomics could offer deeper insights into the spatial and temporal dynamics of METTL3 expression, facilitating more precise diagnostic and therapeutic interventions. Furthermore, rigorous validation studies using large, diverse patient cohorts and longitudinal analyses are essential to establish the reliability and clinical utility of METTL3 as a biomarker. In conclusion, while METTL3 holds promise as a diagnostic marker, its application is fraught with challenges that stem from its context-dependent functions and complex regulatory mechanisms.

The translational potential of METTL3 in clinical applications represents a captivating yet insufficiently explored domain. Preclinical investigations have suggested the therapeutic viability of targeting METTL3 in oncology, autoimmune pathologies, and infectious diseases. Nonetheless, the transition of these findings into clinical practice demands stringent clinical trials to assess the safety and efficacy of METTL3 modulators. A critical focus must be on evaluating off-target effects and the extensive influence of METTL3 on a broad spectrum of downstream mRNA targets. Additionally, understanding patient-specific variations in METTL3 activity could enable personalized therapeutic strategies, optimizing treatment protocols based on individual genetic and epigenetic profiles. The interplay between METTL3 and other components of the m^6^A methylation machinery, such as METTL14, WTAP, and FTO, adds to the complexity, as alterations in these elements can modify METTL3 activity and consequently m^6^A methylation patterns. Moreover, elucidating the role of METTL3 in various immune cells could lead to innovative therapeutic strategies. For instance, investigating how METTL3 regulates the differentiation and function of DCs under different pathological conditions may unveil novel mechanisms of immune modulation that can be targeted in autoimmune diseases or cancer. Similarly, exploring METTL3’s interaction with T cells could reveal new insights into T cell biology. Studying whether METTL3 affects T cell activation, proliferation, and memory formation could potentially identify targets to enhance immune responses against infections or malignancies. Additionally, examining how METTL3 influences the balance between different T cell subsets, such as regulatory T cells and effector T cells, could provide valuable insights into maintaining immune equilibrium and preventing hyperactive immune responses. Furthermore, integrating METTL3 research with advanced methodologies such as single-cell RNA sequencing and CRISPR-Cas9 gene editing could yield high-resolution insights into its cellular and molecular functions. This integration could illuminate the temporal and spatial dynamics of METTL3-mediated modifications and their impact on immune cell behavior. A comprehensive, multidimensional approach that amalgamates molecular, clinical, and technological advancements is essential to fully harness the potential of METTL3 in disease diagnosis and management. Future research should prioritize unraveling the intricate network of interactions involving METTL3 and its downstream targets, thereby paving the way for more precise and effective diagnostic tools.

To address the pressing questions in this field, several cutting-edge techniques can be employed to gain a deeper understanding of METTL3’s role in immune cells and its potential clinical applications. One promising approach is single-cell RNA sequencing (scRNA-seq), which can provide high-resolution insights into the transcriptomic landscape of individual immune cells. By applying scRNA-seq, researchers can identify specific gene expression patterns regulated by METTL3 in various immune cell subtypes, including HSPCs, monocytes, thymic epithelial cells, B, and T cells. This technique can reveal how METTL3 modulates gene expression at a single-cell level, uncovering cell-type-specific regulatory mechanisms that might be overlooked in bulk RNA-seq studies. Another advanced technique is CRISPR-Cas9-mediated genome editing. This technology allows for precise manipulation of the METTL3 gene in specific immune cell populations. By creating knockout or knock-in models, researchers can dissect the functional consequences of METTL3 alterations in different immune cells. For instance, CRISPR screens can be used to identify downstream targets of METTL3 and elucidate their roles in immune cell development, differentiation, and function. Proteomics approaches, such as mass spectrometry-based proteomics, can complement transcriptomic studies by providing a comprehensive view of the protein landscape regulated by METTL3. By comparing the proteomes of METTL3-deficient and wild-type immune cells, researchers can identify differentially expressed proteins and post-translational modifications influenced by METTL3. This information can shed light on the molecular pathways and signaling networks governed by METTL3 at the protein level, offering new insights into its functional roles. Furthermore, advanced imaging techniques like single-molecule fluorescence *in situ* hybridization and live-cell imaging can be utilized to visualize METTL3 localization and dynamics within immune cells. These techniques can reveal how METTL3 interacts with its target RNAs and proteins in real-time, providing spatial and temporal information about its regulatory functions. In addition to these experimental approaches, computational modeling and systems biology can play a crucial role in integrating multi-omics data and predicting METTL3-mediated regulatory networks. By constructing computational models of immune cell signaling pathways and incorporating METTL3-related data, researchers can simulate the effects of METTL3 perturbations on immune cell function and identify potential therapeutic targets.

Overall, this review provides a comprehensive interpretation of the relationship between METTL3 and the immune system, providing novel insights into research gaps that require attention in both research and clinical settings. Furthermore, this review may serve as a valuable reference for researchers and clinicians.

## Supplementary material

The Supplementary Material for this article can be found onlineat: https://www.frontiersin.org/articles/10.3389/fimmu.2024.1456891/full#supplementary-material
