# Supplementary figures and images for: The indispensability of methyltransferase-like 3 in the immune system: from maintaining homeostasis to driving function
Source: Front Immunol. 2024 Oct 2;15:1456891. doi: 10.3389/fimmu.2024.1456891 (PMC11479892; doi:10.3389/fimmu.2024.1456891)

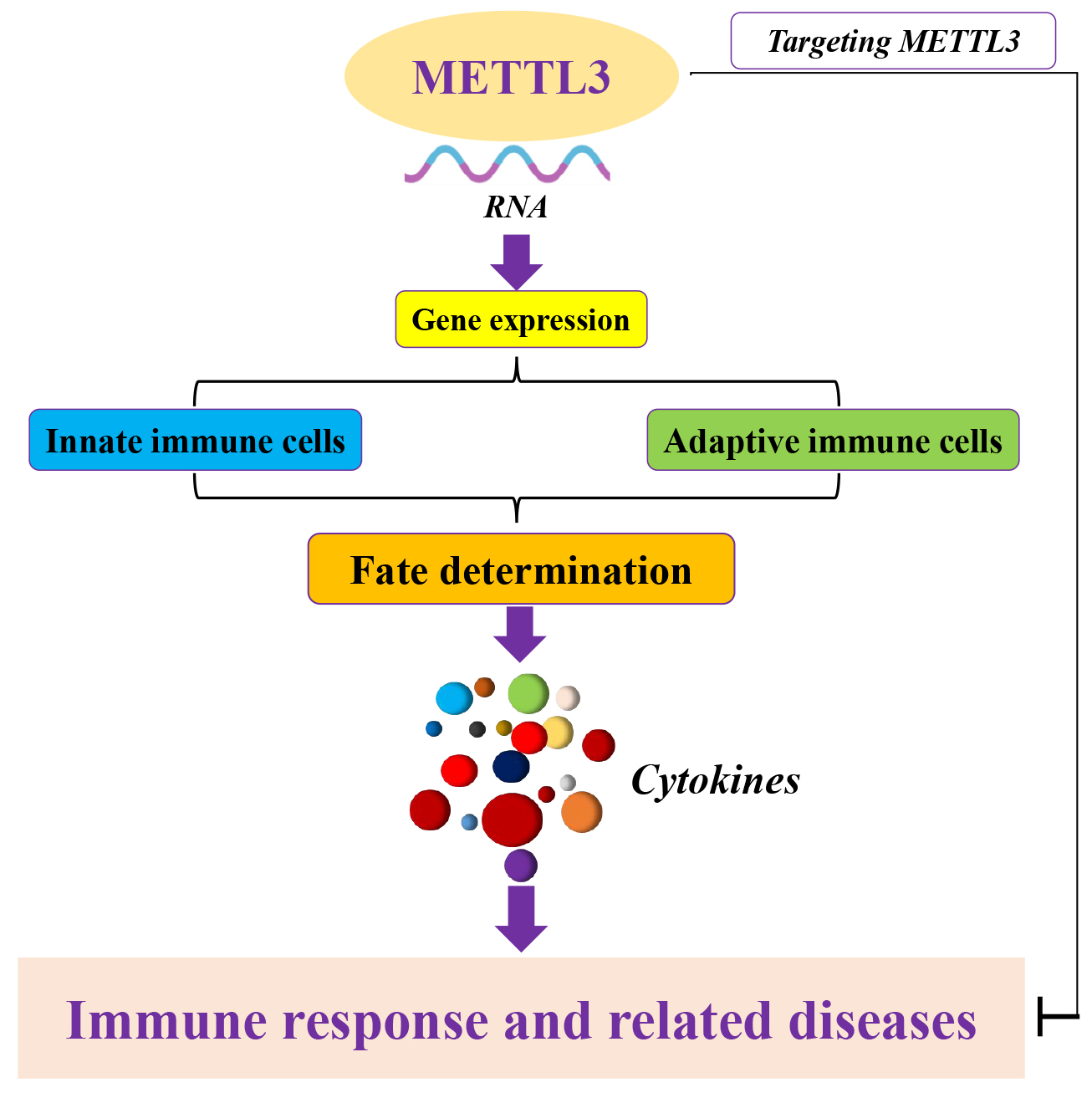

Supplement: Supplementary file 1 [file Image1.jpeg]
